# Neurotransmitters, neuropeptides and calcium in oocyte maturation and early development

**DOI:** 10.3389/fcell.2022.980219

**Published:** 2022-09-23

**Authors:** Maitha M. Alhajeri, Rayyah R. Alkhanjari, Rawad Hodeify, Ali Khraibi, Hamdan Hamdan

**Affiliations:** ^1^ Department of Physiology and Immunology, College of Medicine and Health Sciences and Biotechnology Center, Khalifa University, Abu Dhabi, United Arab Emirates; ^2^ Department of Biotechnology, School of Arts and Sciences, American University of Ras Al Khaimah, Ras Al Khaimah, United Arab Emirates

**Keywords:** calcium, neuronal signal, embryogenesis, oocyte maturation, neurotransmiters

## Abstract

A primary reason behind the high level of complexity we embody as multicellular organisms is a highly complex intracellular and intercellular communication system. As a result, the activities of multiple cell types and tissues can be modulated resulting in a specific physiological function. One of the key players in this communication process is extracellular signaling molecules that can act in autocrine, paracrine, and endocrine fashion to regulate distinct physiological responses. Neurotransmitters and neuropeptides are signaling molecules that renders long-range communication possible. In normal conditions, neurotransmitters are involved in normal responses such as development and normal physiological aspects; however, the dysregulation of neurotransmitters mediated signaling has been associated with several pathologies such as neurodegenerative, neurological, psychiatric disorders, and other pathologies. One of the interesting topics that is not yet fully explored is the connection between neuronal signaling and physiological changes during oocyte maturation and fertilization. Knowing the importance of Ca^2+^ signaling in these reproductive processes, our objective in this review is to highlight the link between the neuronal signals and the intracellular changes in calcium during oocyte maturation and embryogenesis. Calcium (Ca^2+^) is a ubiquitous intracellular mediator involved in various cellular functions such as releasing neurotransmitters from neurons, contraction of muscle cells, fertilization, and cell differentiation and morphogenesis. The multiple roles played by this ion in mediating signals can be primarily explained by its spatiotemporal dynamics that are kept tightly checked by mechanisms that control its entry through plasma membrane and its storage on intracellular stores. Given the large electrochemical gradient of the ion across the plasma membrane and intracellular stores, signals that can modulate Ca^2+^ entry channels or Ca^2+^ receptors in the stores will cause Ca^2+^ to be elevated in the cytosol and consequently activating downstream Ca^2+^-responsive proteins resulting in specific cellular responses. This review aims to provide an overview of the reported neurotransmitters and neuropeptides that participate in early stages of development and their association with Ca^2+^ signaling.

## 1 Introduction

Oocyte maturation and embryogenesis has always been an area of interest for many researchers. Despite the abundant research on the topic, many aspects of the development process are yet to be studied. One specific area of interest is investigating the interplay between neurotransmitters and neuropeptides and oocyte maturation and early development (Herlenius & Lagercrantz, 2001). Similarly, remodeling of Ca^2+^ signaling machinery has been known as a key modulator for oocyte physiology and was carefully studied in several species, including mammals. Based on this, we focus this literature review on the association of neuronal signals with Ca^2+^ in oocyte mutation and early development.

Oocyte maturation is a process by which an immature oocyte arrested at prophase of first meiotic division resumes meiosis to reach competence for normal fertilization after ovulation (Lane et al., 2014). In almost all species examined, oocytes are first arrested at prophase of meiosis I. Under appropriate hormonal control, oocytes proceed in meiotic maturation, to arrest again at metaphase I or metaphase II, depending on the species. At this stage oocytes are known as mature oocytes or eggs and will remain arrested until fertilization (Von Stetina & Orr-Weaver, 2011). During this transition, the oocyte undergoes both nuclear and cytoplasmic maturation that culminate in the formation of the competent egg. Maturation promoting factor (MPF), a complex of a cyclin B and the cyclin-dependent kinase CDK1, is acknowledged as the key stimulator of oocyte maturation. In arrested oocytes, high levels of cAMP inhibit MPF activity. When cAMP decreases, MPF activity is induced and triggers the initiation of germinal vesicle breakdown (GVBD) and chromosome segregation (Adhikari & Liu, 2014).

The role of Ca^2+^ signaling in the oocyte physiology is described in many animal species (Stricker, 1999; Machaca, 2007; Kashir et al., 2013). Studies in mice and frogs demonstrated that extracellular and cytosolic Ca^2+^ are important for meiotic progression. Chelation of extracellular Ca^2+^ and depletion of intracellular Ca^2+^ stores inhibited first-polar-body emission (Tombes et al., 1992; Sun & Machaca, 2004). Remodeling of Ca^2+^ signaling machinery, including Ca^2+^ channels and pumps, is a major element during oocyte maturation and at fertilization and was actively studied in amphibians and mammals (extensively reviewed in (Carvacho et al., 2018)).

**FIGURE 1:**
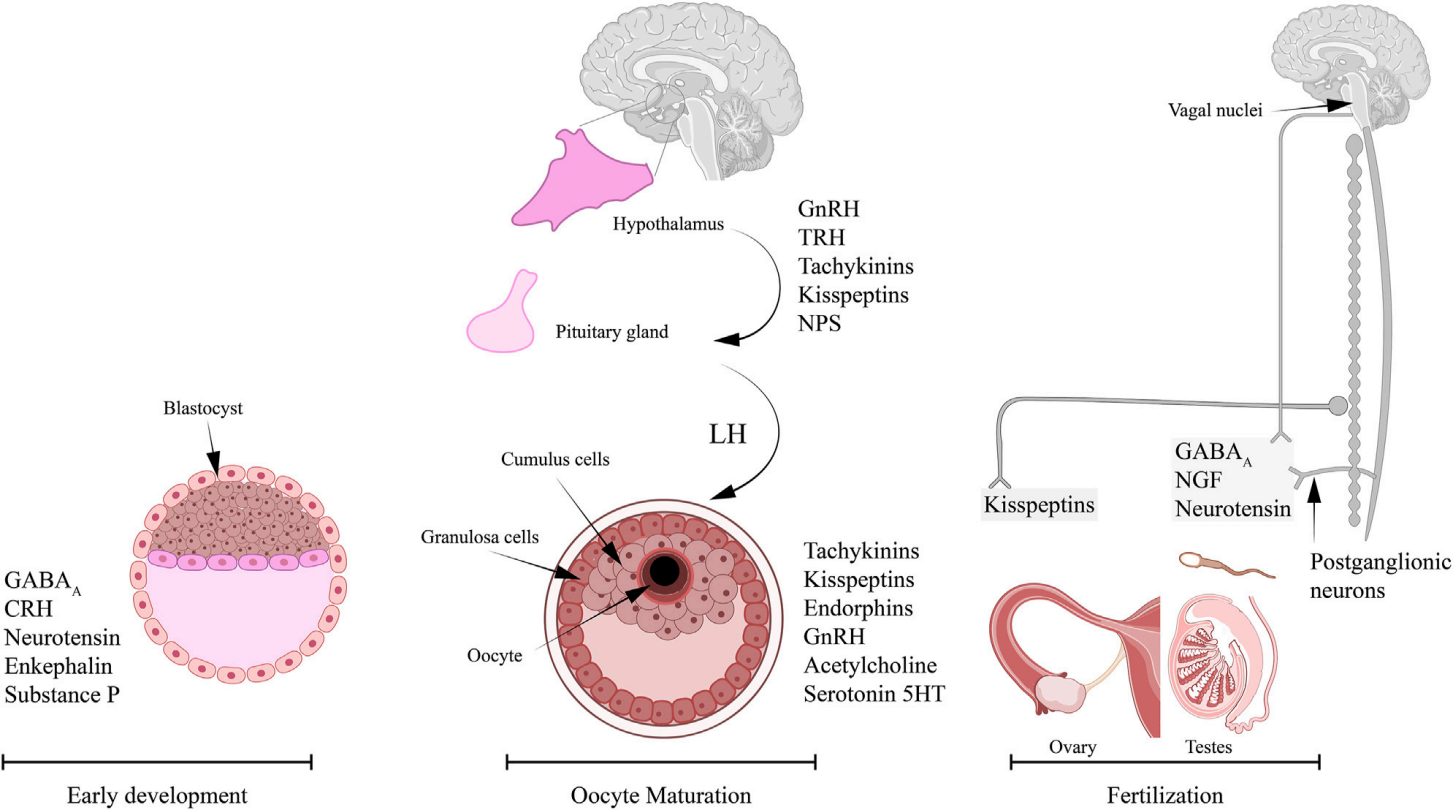
Neurotransmitters and neuropeptides reported to play roles in reproductive processes and dependent on Ca^2+^ signaling. Several studies in vertebrates and invertebrates reported important role for specific neuropeptides in oocyte maturation, sperm activity, and embryogenesis. The neurotransmitters and neuropeptides reported to play an important role in oocyte maturation and involve Ca^2+^ signaling include: tachykinins, kisspeptins, endorphins, GnRH, acetylcholine, and serotonin, 5-HT. The innervation of ovaries is mainly through the nerves from the ovarian plexus. The neuropeptides that are known to regulate sperm activity in a Ca^2+^-dependent mechanism include: GABA_A_, neurotensin (NT), and NGF. It is possible that these neuropeptides are released by the inferior and superior spermatic nerves that innervate the testis. The list of neuropeptides that were associated with early development and involve Ca^2+^ signaling are: GABA_A_, NT, enkephalins, substrate P, and corticotropin-releasing hormone (CRH).

In this review, we will summarize the neurotransmitters and neuropeptides that were known to be associated with Ca^2+^ signaling during oocyte maturation and fertilization (Figure 1). Here, we attempt to collect the available information, to provide an overview of the reported neurotransmitters and neuropeptides that are linked to Ca^2+^ during oocyte maturation. It is worth mentioning that although Ca^2+^ signaling and hormonal regulation is extensively studied in most stages of early development, there is less knowledge on the association between neuronal signaling and Ca^2+^ during key reproductive processes.

Generally, neurotransmitters and neuropeptides are chemical signals that are observed throughout the body, regulating many complicated processes. The function of these chemical signals ranges from controlling autonomic functionalities such as breathing and heart rate to affecting our mood, sleep, and appetite; they alter the body’s response to different stimuli via manipulating electrical signals at the neuronal level. Neurotransmitters are small-molecule chemical messengers such as serotonin, histamine, acetylcholine, glutamate, and gamma aminobutyric acid (GABA). On the other hand, neuropeptides are a specific type of large-molecule neurotransmitters identified as dense-core vesicles for their appearance under electron microscopy (Merighi et al., 2011).

There are two different classes of neurotransmitter receptors, ionotropic and metabotropic. Ionotropic receptors are neurotransmitter-gated ion channels. This class of channels will open after neurotransmitter binding, and messages between neurons using ionotropic receptors will be delivered in less than a millisecond (Sabatini & Regehr, 1996). On the other hand, metabotropic receptors are G protein coupled receptors (GPCR), also known as seven-transmembrane domain receptors, because they activate intracellular signaling proteins called heterotrimeric G proteins. In fact, almost all small-molecule neurotransmitters and most neuropeptides act via metabotropic receptors (Hall, 2004; Beaulieu & Gainetdinov, 2011; Pytliak et al., 2011; Hoyer & Bartfai, 2012; Vaidya et al., 2013; Kruse et al., 2014). By binding to their respective receptors, some reports strongly suggest that neurotransmitters could behave as growth regulators during specific developmental periods, and are prominent candidates for transcellular signals that could stimulate the development of central nervous system (CNS) cells, since they surround neural cells throughout the CNS maturation period (Cicirata et al., 1991; Miranda-Contreras et al., 1998; Miranda-Contreras et al., 1999; Miranda-Contreras et al., 2000) (Cameron & Gould, 1994; LoTurco et al., 1995; Wang et al., 1996; Antonopoulos et al., 1997; Lauder et al., 1998; Ma et al., 1998; Weiss et al., 1998; Butler et al., 1999; Fiszman et al., 1999; Brezun & Daszuta, 2000; Haydar et al., 2000; Ma et al., 2000). It is now established that specific neurotransmitter receptors are present on progenitor cells of the developing CNS and could play, during neural development, a role that has remained unidentified until recently (Hodo et al., 2020; Xing & Huttner, 2020).

## 2 Neuronal signaling in oocyte maturation and embryogenesis

Different signaling pathways have been recognized as vital regulators of early organism development, such as fibroblast growth factor (FGF), Hedgehog, Wnt, transforming growth factor-β (TGF), and Notch (Sanz-Ezquerro et al., 2017). These signals are usually transduced via chemical or hormonal messengers. On the other hand, several studies have succeeded in illustrating the role of various neurotransmitters and neuropeptides in processes like oocyte maturation and embryo development. Below we sought to review the neurotransmitters and neuropeptides that were reported to play a role in oocyte maturation and early development in both vertebrates and invertebrates and present known connections to Ca^2+^ signaling during these important processes.

### 2.1 Neuronal innervation of the gonads

In mammals, the gonads of both males and females and other reproductive organs are innervated by the parasympathetic and sympathetic efferents (Louis et al., 1978). A growing of evidence suggests that the gonads are regulated by the autonomic nerves. It has also been suggested that the cerebral structures that are connected to the gonads control the secretion of hormones. This suggests that the gonads are independent of the brain’s neurodevelopment. (Gerendai et al., 2005). The vagal innervation plays an important role in the development of the ovarian control. It has been known that vagotomy can delay the onset of puberty and decrease the number of ova shed (Ojeda et al., 1983; Nakamura et al., 1992).

Innervation of the ovaries is mainly through the nerves from the ovarian plexus. Most of these nerves are noradrenergic nerves (D'Albora et al., 2000; Lawrence & Burden, 1980). Some studies showed that primate ovaries contain groups of catecholaminergic neuron-like cells expressing nerve growth factor receptors (Dees et al., 1995). On the other hand, the inferior and superior spermatic nerves innervate the testis. The superior spermatic nerve is the main nerve to the innervation of the testis (Setchell et al., 1994). The parasympathetic portion of the spermatic nerve is composed of the vagus nerve. The sympathetic fibers of the nerve are derived from the hypogastric and mesenteric plexuses, while the more parasympathetic ones are derived from the pelvic nerve (Nagai et al., 1982; Gerendai et al., 2005).

### 2.2 Neuronal signaling in oocyte maturation

Oocyte maturation is a required process in the completion of female oogenesis to produce mature eggs suitable for fertilization and embryogenesis in both vertebrates and invertebrates (Deguchi et al., 2011; Lane et al., 2014). In echinoderms, amphibians, and mammals, the oocytes are initially arrested in prophase of meiosis I before released to continue first division and become arrested again at the metaphase of meiosis II (in mammals and frogs) whereas in echinoderms they are blocked later, at the interphase stage (Picard et al., 1987; Kishimoto, 1998). It is known that hormonal signaling initiated by luteinizing hormone (LH) or gonad-stimulating hormones as well as environmental factors modulate a diverse signaling pathway resulting in activation maturation promoting factor (MPF) (Jessus et al., 2020). Although less clearly understood compared to hormonal signaling, the role of the neuronal signaling in oocyte maturation is reported in both vertebrates and invertebrates, including mammals, insects, amphibians, fish, and echinoderms (Sullivan & Levin, 2016). Sheng et al. demonstrated that through G protein-coupled receptor, serotonin causes meiosis arrest in both frogs and mice (Sheng et al., 2005). While in other amphibians and starfish, serotonin has been implicated in resumption of oocyte maturation (Buznikov et al., 1993; Stricker & Smythe, 2001). Zhang et al. identified the role of the brain-derived neurotrophic factor (BDNF) in oocyte maturation via protein kinase B transduction pathway (Zhang et al., 2010). Upon stimulation by luteinizing hormone (LH), BDNF can be secreted by granulosa and cumulus cells in the ovaries to act on its receptors, TrkB receptors, expressed in oocytes to mediate first polar body extrusion (Kawamura et al., 2005). Kato et al. using sea cucumber, *Apostichopus japonicus*, injected two neuropeptides, NGIWYamide and NGLWYamide into the abdominal cavities of sexually mature males and females, which resulted in induction of gamete spawning. Despite these valuable findings, the exact sites of action and synthesis of NGIWYamide are still unknown. The study was only able to illustrate the importance of the ovarian wall for the function of the peptide (Kato et al., 2009). Furthermore, some studies have discerned that in addition to inducing oocytes maturation, neuronal signaling can also maintain their quiescence as a strategy ensuring the continuity and survival of a species during times of food or mates’ scarcity (Kim et al., 2021). Kim et al. clarified the role of noradrenergic signaling in this evolutionary strategy in four species; *Caenorhabditis elegans*, *Caenorhabditis remanei*, *Drosophila melanogaster*, and *Danio rerio*, where the two identified neurotransmitters were norepinephrine (NE) and octopamine (OA) (Kim et al., 2021).

Different hormonal signals have been recognized for their vital role in the various reproductive processes, such as ovulation. Oocyte maturation in vertebrates is triggered by luteinizing hormone (LH) surge secreted by the anterior pituitary in response to the pulsatile release of gonadotropin releasing hormone (GnRH) by the hypothalamus (Voronina & Wessel, 2003; Abbara et al., 2018; Dufour et al., 2020). However, few studies elaborated on the role of neurotransmitters and neuropeptides during ovulation. The neuropeptide neurotensin (NT) was first identified in bovine hypothalamus (Carraway & Leeman, 1973) and was reported to be expressed in other species and tissues, including humans and rat ovaries during the periovulatory period (Al-Alem et al., 2021). Soon several reports demonstrated role for NT in multiple aspects of ovarian function and sperm capacitation (Reinecke, 1987; Dissen et al., 1996; Hiradate et al., 2014; Cerny et al., 2015; Sakumoto et al., 2015; Umezu et al., 2016). NTS was detected in both theca and granulosa cells of follicles. Ferris et al. showed that NT stimulate release of LH in the rat (Ferris et al., 1984). Expression of NT was induced by human chorionic gonadotropin (hCG) in the granulosa cells (GC) of human and rat ovary prior to ovulation (Wissing et al., 2014). It was also demonstrated that NT bind mainly to non-G protein coupled receptor, sortilin receptor 1 (SORT1), which is the predominant NT receptor found in human and rat GCs (Al-Alem et al., 2021).

### 2.3 Neuronal signaling in embryogenesis

Neuronal signaling plays an imperative role in embryo development and early organogenesis. One of the earliest neurotransmitters involved in embryo development are catecholamines; they include noradrenaline, adrenaline, and dopamine. Generally, this class of signaling molecules can modulate emotions, memory, actions and attitudes, hormonal balance, and cardiovascular processes (Herlenius & Lagercrantz, 2001; Jiang et al., 2020). However, studies demonstrated that noradrenaline is specifically essential for the maturation of the cerebral cortex, and dopamine is necessary for movement coordination (Herlenius & Lagercrantz, 2001). Dopamine mainly exerts its effects on metabotropic receptors. There are five dopamine receptors categorized into two classes, D1-like and D2-like dopamine receptors. It has been reported that D1-like signaling, mainly through stimulation of coupled G*α*_s_ and G*α*_olf_ (Tesmer et al., 1997) enhances the activity of adenylate cyclase in the retina (Brown & Makman, 1972) and in rat neostriatum (Kebabian et al., 1972). Another signaling pathway for D1-like is demonstrated in *Xenopus* oocytes and is dependent on phospholipase C-mediated IP3 production followed by IP3-dependent mobilization of intracellular Ca^2+^ (Mahan et al., 1990). It has been also reported that dopamine receptors stimulation modulates several ion channels including high-voltage-activated Ca^2+^ currents in several types of vertebrate and invertebrate neurons *in vitro* (Paupardin-Tritsch et al., 1985; Marchetti et al., 1986; Surmeier et al., 1995; Hernández-López et al., 1997). D2-like signaling can have antagonistic effects on adenylate cyclase depending on the coupled G protein subunits. D2-like signaling, through G*α*_i_, was shown to inhibit adenylate cyclase activity in several cell types (Huff et al., 1998; Oak et al., 2000). Other studies reported that G protein *βγ* subunits released by D2-like receptor signaling can have stimulatory effects on other adenylate cyclase such as adenylate cyclase 2 and 4 free G*βγ* (Taussig et al., 1994; Sunahara et al., 1996; Watts & Neve, 1997). In chinese hamster ovary cells, D2-like signaling via G*βγ* proteins stimulates phospholipase C*β*1, causing inositol trisphosphate-induced Ca^2+^ mobilization and reduction in L-type Ca^2+^ currents (Kanterman et al., 1991).

Another molecule that appears early in the embryonic stage is serotonin, 5-HT, a neurotransmitter that falls under the biogenic amines category. Usually, its signal is transduced via different metabotropic receptors, except 5-HT3, an ionotropic receptor (Sahu et al., 2018). Serotonin has been involved in various processes both at the cellular and systemic levels. Such cellular processes include apoptosis, mitochondrial biogenesis, cell proliferation, and migration (Sahu et al., 2018). However, its systemic effects involve modulating different biological activities within and outside the CNS. Within the CNS, many studies illustrated the role of this neurotransmitter in memory, mood regulation, and social cognition (Sahu et al., 2018). Outside the CNS, serotonin can regulate metabolism by regulating glucose and lipid metabolism. Also, some reports confirm its role in controlling rhythmic breathing (Sahu et al., 2018). Moreover, several *in vitro* studies illustrated how crucial serotonin is in early development (Herlenius & Lagercrantz, 2001). For example, it is involved in the normal morphogenesis of the heart and craniofacial epithelia. Serotonin-producing cells are among the first cells to be generated in the brain, demonstrating the importance of adequate amounts of serotonin in the normal development of the brain, specifically, the somatosensory cortex (Herlenius & Lagercrantz, 2001).

Additionally, acetylcholine (Ach) is another important neurotransmitter that plays a significant role in regulating the immune system as it controls inflammation by preventing specific inflammatory cells’ effects in non-neuronal cells (Jiang et al., 2020). Moreover, it is formed at later stages of embryo development. Like noradrenaline, its presence at normal levels has been associated with the development of a normal cerebral cortex, and anomalies in its levels can lead to slow comprehension and reduced mental capabilities (Herlenius & Lagercrantz, 2001). Acetylcholine is associated with two primary receptors, which are nicotinic and muscarinic (Jiang et al., 2020). The muscarinic receptors are G-protein coupled receptors linked to either G_q_ or G_i_ (Jiang et al., 2020). Ach is synthesized in both neuronal and non-neuronal cells, and the role it plays in the body is highly dependent upon its site of synthesis.

GABA, a classical amino acid neurotransmitter, is considered the primary inhibitory signal in the CNS of adult animals (Herlenius & Lagercrantz, 2001). As an inhibitory transmitter, GABA contributes to functions like producing smooth movements and modulating respiratory rate (Jewett & Sharma, 2022). Its effect is elicited through either the ionotropic receptor, GABA_A_, or the metabotropic receptor, GABA_B_. GABA_A_ receptor is a chloride ion channel that when activated, results in cell membrane hyperpolarization. In contrast, GABA_B_ is a G-protein coupled receptor that decreases presynaptic Ca^2+^ conductance and increases postsynaptic K^+^ conductance (Jewett & Sharma, 2022). GABA plays a pivotal role in regulating brain development in embryogenesis, via binding to both types of receptors (Herlenius & Lagercrantz, 2001). It also plays key role in the migration and maturation of neuronal precursors during neural development (Behar et al., 1994; Behar et al., 1996; Behar et al., 2000).

Another neuropeptide hormone, corticotropin-releasing hormone (CRH), was reported to play a role in promoting synapse formation, cell survival, and plasticity, specifically in the olfactory bulb. The study by Garcia et al. revealed that manipulating the CRH signaling levels can affect the formation of synaptic proteins and the maintenance of active synapses. (Garcia et al., 2014).

### 2.4 Neuronal signaling and calcium

Neuronal signaling is highly dependent on the levels of Ca^2+^ concentrations. Eshra et al. studied the effects of different Ca^2+^ concentration levels on vesicle priming, fusion, and replenishment at mossy fiber synapses in the rodent cerebellum. This study showed Ca^2+^ dependent vesicle fusion*,* as demonstrated in previous studies. In terms of vesicle priming, the results indicated the presence of a high-affinity Ca^2+^ sensor at the studied synapse that initiated the process of priming at intracellular Ca^2+^ concentrations in the range of 30 and 180 *nM*. Finally, there is a low association between intracellular Ca^2+^ concentration and vesicle recycling. Overall, the study illustrated the high dependence of the neuronal signaling process, specifically the phases of priming and fusion, on the intracellular Ca^2+^ levels (Eshra et al., 2021).

The role of the neuropeptide S (NPS) and NPS receptor 1 (NPSR1) complex in activating the intracellular pathways of Ca^2+^ release has been under investigation. In an *in vitro* study utilizing a viral vector, experiments were designed to involve an adenovirus vector that included the gene to express NPSR1, and the vector was then delivered to cultured mouse hippocampus neurons. Further, the Ca^2+^ levels were visualized using extracellular solutions and a laser scanning microscope. To examine the visuals, the experimenters used different data analysis tools. The significant findings of this study were that the NPS-NPR1 complex induced the release of both intracellular and extracellular Ca^2+^. The authors found that the phosphorylation of PIP_2_ into DAG and IP_3_ by phospholipase C (PLC) mainly regulated intracellular Ca^2+^ release, which led to the activation of IP_3_ receptors and, subsequently, the ryanodine receptors attached to the endoplasmic reticulum, increasing Ca^2+^ concentrations in the cytosol. Moreover, to exclude the presence of other pathways triggered by NPSR1 activation, the researchers incorporated into the culture receptor-specific and PLC blockers and noted that the Ca^2+^ concentration levels did not increase, thereby concluding that NPSR1 mediates intracellular Ca^2+^ release only through G_q_ signaling (IP_3_-DAG Pathway). Overall, several studies link the NPSR1 gene to the development of panic and anxiety disorders. Thus, the authors indicated that their study design and findings will benefit future research in developing a better understanding of the clinical implications of the gene in anxiety and panic disorders (Erdmann et al., 2015).

Homma et al. demonstrated an important role for Ca^2+^ signaling in serotonin (5-HT) mediated neurite outgrowth (Homma et al., 2006). Using PC-12 cells as a model of neuronal differentiation, the authors demonstrated that treatment with 5-HT enhanced nerve growth factor (NGF)-induced neurite outgrowth. The study also indicated that 5-HT through HT_3_ receptors activate L-type Ca^2+^ channels leading to rise in intracellular Ca^2+^, and subsequent activation of Ca^2+^ dependent proteins, calmodulin and calcineurin. In a different study using same model of PC-12 cells, Gysbers et al. (2000) reported enhancement of NGF-induced neurite outgrowth by extracellular guanosine 5′ triphosphate (GTP). This study demonstrated that GTP induced intracellular Ca^2+^ rise is mediated through L-type Ca^2+^ channels and Ca^2+^-induced Ca^2+^ release from intracellular stores (Gysbers et al., 2000). In another study, Rondé and Nichols showed that 5-HT through HT_3_ receptors activate presynaptic voltage-gated Ca^2+^ channels leading to Ca^2+^ influx and enhancing exocytosis in a subset of striatal brain nerve terminals which can affect release of neurotransmitters (Ronde & Nichols, 1998).

An interesting study by Irwin and Allen tested the association of Ca^2+^ signaling and the effect of several neuropeptides in the modulation of gene expression by light in the suprachiasmatic nucleus (SCN) neurons (Irwin & Allen, 2010). The results demonstrated that two of the neuropeptides expressed in SCN neurons, vasoactive intestinal peptide, and arginine vasopressin, regulate Ca^2+^ homeostasis, leading to regulation of SCN neuronal synchronization.

Among the critical cellular functions controlled by the Ca^2+^ signal is the regulation of gene expression. Different extracellular signaling molecules can trigger an increase in free intracellular Ca^2+^ that, in turn, can induce gene transcription. In the CNS, some neuronal signals can regulate Ca^2+^-triggered gene expressions necessary for various neuronal activities such as synaptic plasticity, memory formation, neurogenesis, and neuroprotection. Lobos et al. assessed the role of neural stimulation of hippocampal neurons in triggering Ca^2+^- dependent gene transcription via RyR-mediated Ca^2+^ release. Blocking GABA_A_ receptors using gabazine (GBZ) promoted a synchronic, transient increase in Ca^2+^ in the nucleus of hippocampal neurons. This increase translated into elevated levels of phosphorylated CREB, Nps4, and RyR2 mRNA. This study proved the imperative role of RyR2 and Ca^2+^ induced- Ca^2+^ release in the generation of nuclear Ca^2+^ transients following neuronal stimulation either through GBZ or glutamate uncaging (Lobos et al., 2021).

### 2.5 Neuronal signaling and calcium regulation during oocyte maturation and fertilization

Meiotic arrest during oogenesis is common in both vertebrates and invertebrates to ensure timely preparation of the oocyte to achieve full competency for fertilization (Von Stetina & Orr-Weaver, 2011). In most vertebrates, oocytes primarily arrested in prophase of meiosis I, resume meiosis and undergo germinal vesicle breakdown to produce mature oocytes that arrest at metaphase of the second meiotic division until fertilization (Von Stetina & Orr-Weaver, 2011; Nader et al., 2013). Meiotic arrest and resumption are controlled by specific molecular events (Machaca, 2007; Santella et al., 2020; Hatirnaz et al., 2022; Mostafa et al., 2022), and are driven by a complex cascade of hormonal signaling (Abbara et al., 2018; Stewart & Davis, 2019). Despite the complexity and diversity of these molecular and physiological events, they converge on Ca^2+^ as a key orchestrator of these developmental events (Figure 1).

The signals for maintaining oocyte arrest were recently revisited in the study by Kim et al. who investigated the role of noradrenergic signaling in this reversible quiescence. This study demonstrated that the ovaries of four species, *Caenorhabditis elegans*, *Caenorhabditis remanei*, *Drosophila melanogaster*, and *Danio rerio,* were heavily innervated by cells producing noradrenergic molecules, specifically Octopamine (OA) or Norepinephrine (NE). In *Caenorhabditis*, the pivotal role of OA in maintaining oocytes’ quiescence was proven through mutant nematodes lacking endogenous OA. The study identified SER-3, a G-protein-coupled receptor (G_*q*_), as the receptor targeted by OA and allowing the transduction of downstream inhibitory signals. In *Drosophila melanogaster*, the balance between this noradrenergic and the nutrient signals determines the onset of oogenesis. Mutant forms of *Drosophila melanogaster* revealed the importance of OA in inhibiting the progression of egg chambers during nutrient insufficiency. This action, in turn, is mediated via either alpha or beta-adrenergic receptors. Like *Drosophila melanogaster*, the maintenance of oocyte quiescence in *Danio rerio* females is governed by the balance between the nutrient and noradrenergic signals. The only difference is that the ovaries in this species are innervated by NE-producing cells (Kim et al., 2021).

At the time of ovulation, gonadotropin-releasing hormone (GnRH) acts on the pituitary gonadotrophs to induce biphasic Ca^2+^ rise, resulting ultimately in luteinizing hormone (LH) release (Lanzone et al., 1989). The Ca^2+^ spike phase reflect release from internal stores while the plateau phase is believed to be caused by Ca^2+^ influx via voltage sensitive Ca^2+^ channels (McArdle et al., 1996). Other studies have also shown LH-independent role for GnRH in oocyte maturation through other aspects, such as stimulation of follicle enclosed oocytes (Hillensjo & LeMaire, 1980), and induction of prostaglandin synthesis by granulosa cells (Clark et al., 1980). In addition to stimulatory function on oocyte maturation, GnRH can have modulatory ovarian function by inhibiting the action of the follicle stimulating hormone (FSH) in immature follicles and by suppressing gonadotropin action in mature follicles. These processes were also dependent on Ca^2+^ signaling associated with phospholipids breakdown and activation of Ca^2+−^dependent protein kinase C (PKC) (Knecht et al., 1985).

Like GnRH, thyrotropin releasing hormone (TRH) is another peptide that can modulate pituitary secretion and was shown to trigger a biphasic Ca^2+^ dynamic in oocytes expressing rat TRH receptors (Eidne et al., 1994). TRH receptors are G-protein-coupled receptors that were detected in human, rodents, and frogs (Sun et al., 2003). TRH-mediated signaling initiate inositol phosphate and diacylglycerol generation, leading to Ca^2+^ mobilization and activation of PKC, respectively (Gershengorn, 1986; Naor, 1990). Shapira *et al* nicely demonstrated that although both acetylcholine and TRH trigger inositol 1,4,5-trisphosphate (IP_3_) generation, they induce Ca^2+^ mobilization from distinct cellular stores in the *Xenopus* oocyte, suggesting that these different compartments are associated with different receptors (Shapira et al., 1990). Unfortunately, these studies did not investigate the effect of TRH on oocyte maturation.

Tanabe et al. studied the association between Ca^2+^ signaling and the neurotransmitter serotonin (5-HT), in triggering oocyte maturation, spawning, and early development in bivalve mollusks (Tanabe et al., 2006). By binding to surface receptors on oocytes, serotonin induced cytosolic Ca^2+^ levels. The authors demonstrated an important role for extracellular Ca^2+^ in 5-HT induced oocyte maturation. Oocytes incubated in a Ca^2+^-free environment failed to mature despite the presence of 5-HT. Furthermore, this study identified a novel neural factor, named oocyte maturation arresting factor (OMAF), that can suppress 5-HT-induced oocyte maturation. The factor was detected with serotonin-containing neurons in the cerebral and pedal ganglia in the CNS of these bivalve mollusks (Tanabe et al., 2006).

Tachykinins and kisspeptins are neuropeptides that are secreted by terminal sensory neurons and discrete populations of hypothalamic neurons and (Garcia-Ortega et al., 2016) and were reported as important regulators of reproduction including GnRH secretion (Lehman et al., 2010; Clarke et al., 2015). The study by García-Ortega et al. showed that both tachykinins and kisspeptins signaling pathways are active in human mural granulosa and cumulus cells (Garcia-Ortega et al., 2016). Interestingly, the two systems function in coordination to control granulosa cell function in the human ovary. This study demonstrated that peptide kisspeptin caused an increase intracellular Ca^2+^ in these cells while tachykinin signaling reduced Ca^2+^ mobilization, suggesting an essential role for Ca^2+^ in this process. Ca^2+^ mobilization during oocyte maturation was also reported with Ach, a neurotransmitter released by peripheral cholinergic neurons. Acetylcholine was shown to promote progesterone-induced oocyte maturation through Ca^2+^ release from cellular stores and is independent of Ca^2+^ influx from the extracellular space (Gelerstein et al., 1988).

Endorphins, opioid neuropeptides produced in the brain and peripheral nervous system, arrested bovine oocytes at metaphase I when cultured in hormone-free medium. Applying low levels of antagonist to μ-opioid receptor expressed in cumulus-oocyte complexes and mural granulosa cells, reversed the effect of endorphins, and resulted in increasing the rate of oocytes arrested in metaphase I. The effect was abolished in the presence of Ca^2+^ chelator, BAPTA-AM, demonstrating an important role for Ca^2+^ rise in the cumulus-oocyte coupled signaling associated with oocyte maturation (Dell'Aquila et al., 2002).

A study by Erdmann et al., 2015 investigated the role of the NPS and NPSR1 complex synaptic plasticity and its connection to Ca^2+^ signaling. Expression of NPSR1 in primary mouse hippocampal neurons led to release of Ca^2+^ from intracellular stores and activated store-operated Ca^2+^ entry (Erdmann et al., 2015). Although no studies were reported to investigate NPS direct effect on oocyte maturation, administration of NPS was found to modulate hypothalamic-pituitary-adrenal axis activity (Smith et al., 2006). Although NPS is mainly abundant in brain, the NPS–NPSR1 system was detected in testis and other endocrine glands and is found in all vertebrates except fish (Reinscheid, 2007). The neuropeptide S receptor (NPSR) is a member of G-protein coupled receptor (GPCR) superfamily (Reinscheid & Xu, 2005; Okamura et al., 2008; Ruzza et al., 2010). In vertebrate oocytes, active GPCRs coupled to Gα_s_ were associated with arrest in prophase of meiosis I, through induction of adenylate cyclase and maintaining high cytoplasmic cyclic adenosine monophosphate (cAMP) concentrations (Kalinowski et al., 2004; Freudzon et al., 2005; Nader et al., 2014). Surprisingly, in oocytes of several invertebrates show that increase in cytoplasmic cAMP concentration is required for meiotic maturation (Deguchi et al., 2011). Other studies showed that activation of GPCR and uncoupling of G beta gamma dimer regulates the activity of PLC in frog oocytes (Stehno-Bittel et al., 1995). Carnero et al. showed that injection of PLC *into Xenopus laevis* oocytes induced oocyte maturation in these cells (Carnero & Lacal, 1993).

On the other hand, neuropeptides association with Ca^2+^ signals were also reported in the interaction of sperm with mature oocyte. Kuroda et al. reported that GABA_A_ signaling increased cytosolic Ca^2+^ and promoted progesterone-induced acrosomal reaction in mammalian sperm (Kuroda et al., 1999). In other studies, in mice and bovine spermatozoa, the neurotransmitter NT was reported to induce cytosolic Ca^2+^ and enhance acrosomal reaction and the capacity of the sperm during fertilization (Hiradate et al., 2014; Umezu et al., 2016). Some studies demonstrated that NGF binding to neurotrophin (Trk) receptors expressed in mammalian sperms promote sperm motility through the female genital tract and enhances acrosomal reaction (Jin et al., 2010), while other studies showed NGF enhanced sperm survival without affecting both Ca^2+^ levels and acrosome reaction (Li et al., 2010). See excellent review by Ramirez-Reveco et al. on sperm functionality and neuronal signaling (Ramirez-Reveco et al., 2017). In a separate study in bovine sperm, Etkovitz et al. demonstrated that activation the epidermal growth factor receptor (EGFR) via the epidermal growth factor (EGF) evoke rise in intracellular Ca^2+^ and promoted acrosomal reaction (Etkovitz et al., 2009).

Similarly, Ca^2+^ plays critical role during neuronal signaling during early development. In the CNS, some neuronal signals can regulate Ca^2+^- triggered gene expressions necessary for various neuronal activities such as synaptic plasticity, memory formation, neurogenesis, and neuroprotection. Blocking GABA_A_ receptors promoted a synchronic, transient increase in Ca^2+^ in the nucleus of hippocampal neurons. This increase translated into elevated levels of phosphorylated cAMP response element (CREB) and Neuronal Per Arnt Sim domain protein 4 (Npas4) (Pegoraro et al., 2010; Mauceri et al., 2011; Bengtson et al., 2013). On the other hand, Ca^2+^ is involved in axis induction and dorsoventral patterning in vertebrates, such as *Xenopus* and zebrafish, via interaction between canonical and noncanonical Wnt pathways. Ca^2+^ release has also been associated with the orchestration of the morphogenetic movements during organogenesis (Slusarski & Pelegri, 2007). Sato et al. showed that NT-dependent Ca^2+^ mobilization was also associated with early developmental stages in primary cultures of cerebral cortex cells from neonatal rats (Sato et al., 1991). Additional neuropeptides, including GABA_A_, enkephalin, and substrate P, have been detected in the developing brain at early stages and were shown to be induced together with Ca^2+^-binding proteins during fetal development (Kwong et al., 2000).

## Conclusion

Several neurotransmitters including neuropeptides are known to play a role in oocyte maturation and early development in vertebrates and invertebrates. Examples are noradrenergic molecules, serotonin, neuropeptides coupled to G-protein coupled receptor, GABA, and dopamine. However, few of these neurotransmitters were demonstrated to be dependent on Ca^2+^ changes in modulating processes during oocyte maturation and early development. Further studies are needed to understand the molecular machinery modulated by Ca^2+^-dependent neuropeptides signaling. Equally important is to identify new gonadal neuropeptides and their receptors. Understanding the role of neuropeptide signaling in abnormalities associated with fertility and early development could pave the way for targeting these pathways during therapeutic interventions.
